# Prediction of cervical cancer screening: application of the information-motivation-behavioral skills model

**DOI:** 10.1186/s12885-024-12098-9

**Published:** 2024-03-19

**Authors:** Marzieh Ghasemi, Mitra Savabi-Esfahani, Mahnaz Noroozi, Mohammad Satari

**Affiliations:** 1 Department of Midwifery, Faculty of Medical Sciences, Shahrekord Branch, Islamic Azad University, Shahrekord, Iran; 2 Student Research Committee, Isfahan University of Medical Sciences, Isfahan, Iran; 3 Department of Midwifery and Reproductive Health, Faculty of Nursing and Midwifery, Isfahan University of Medical Sciences, Isfahan, Iran; 4 Department of Midwifery and Reproductive Health, School of Nursing and Midwifery, Isfahan University of Medical Sciences, Isfahan, Iran; 5 Department Health Information Technology, Health Information Technology Research Center, Isfahan University of Medical Sciences, Isfahan, Iran

**Keywords:** Motivation, Behavior, Cervical cancer, Screening

## Abstract

**Introduction:**

Screening is an effective method for preventing cervical cancer. The present study aimed to determine the predictability of cervical cancer screening using the information-motivation-behavioral skills (IMB) model, as this model can help understand the factors that influence health-related behaviors.

**Method:**

The present cross-sectional study examined 310 women aged 20 to 60 in Isfahan, Iran, between 2020 and 2021. To this end, comprehensive health centers and gynecology clinics of hospitals were randomly selected by lot. Women who met the study’s inclusion criteria were selected via convenience sampling. An IMB skills questionnaire developed by researchers comprised the data collection tool. The data were analyzed using SPSS 22 software, descriptive and regression tests, and AMOS 24.0 software.

**Findings:**

Approximately 18.1% of the participants had never undergone routine cervical cancer screening. The regression model results indicated that the model components accurately predicted regular cervical cancer screening (*P* < 0.00). Path analysis revealed that information (β = 0.05, *P* = 0.002), motivation (β = 0.187, *P* = 0.026), and behavioral skills (β = 0.95, *P* < 0.001) were directly associated with regular cervical cancer screening. Furthermore, behavioral skills had the greatest direct effect on regular cervical cancer screening.

**Discussion and conclusion:**

The results demonstrated that the IMB model accurately predicted cervical cancer screening. Therefore, it is possible to improve cervical cancer screening in women by designing and implementing interventions based on this model’s components, particularly those that improve behavioral skills.

**Supplementary Information:**

The online version contains supplementary material available at 10.1186/s12885-024-12098-9.

## Introduction

Cervical cancer is the fourth most prevalent cancer in women worldwide [1] and the second most common cancer of the reproductive tract after ovarian cancer in Iran [2]. The incidence and mortality rates of cervical cancer are significantly higher in regions with a low or moderate Human Development Index (HDI). Meanwhile, between 84 and 90% of cancer deaths occur in low- and middle-income countries [3]. Advanced cervical cancer is one of the leading causes of death from this cancer [4], whereas this disease is curable if detected in its precancerous stage [5]. Early cancer detection through screening is an important factor in reducing cancer-related mortality. Screening is a cost-effective and potent method of preventing cervical cancer [6, 7], and this method has been recommended, particularly for countries with high morbidity and mortality rates [8]. The program for cervical cancer screening aims to conduct screening at appropriate and regular intervals [9]. According to studies, regular screening reduces the risk of dying from cervical cancer [10, 11]. Regular screening is recommended from the age of 21 to 65 for women who are sexually active [12]. According to Iran’s national guidelines, cervical cancer screening is performed in women aged 30 to 59 years, and if a person is under 30 or over 59 years of age, or in the intervals between regular examinations, there are suspicious symptoms of cervical cancer (abnormal bleeding, pain during sexual intercourse and foul-smelling discharge from the genital tract) should perform more complete evaluations to confirm or rule out cervical cancer [13]. The Healthy People 2020 program recommends that at least 93% of women undergo regular cervical cancer screening [14].

Despite the recommendation, only 60% of women in developed countries and 20% of women in developing countries undergo the Pap smear test, according to studies [8, 15]. Moreover, women are not routinely screened in certain countries [16]. Mohebi et al. [17] reported that only 11.25% of Iranian women routinely received a Pap smear.

In Iran, the cervical cancer prevention program is implemented on an opportunistic foundation [18]. The aim of the preformation of the cervical cancer prevention program is early diagnosis and screening of cervical cancer, identification of people suspected of or suffering from cervical cancer, and then the treatment and care of patients [13].

According to a systematic review, less than 45% of Iranian women had undergone a Pap smear test at least once. Approximately half of the Iranian women were aware of cervical cancer and the Pap smear test [19]. Izadi et al. [20] revealed that only 34% of women participated in cervical cancer screening.

Using behavior change patterns can be extremely beneficial in identifying and predicting cervical cancer screening factors. To this end, a theory that is effective in predicting behavior, mediating factors that affect behavior, and measures that result in behavioral change should be selected to examine health behaviors [21].

**Fig. 1:**
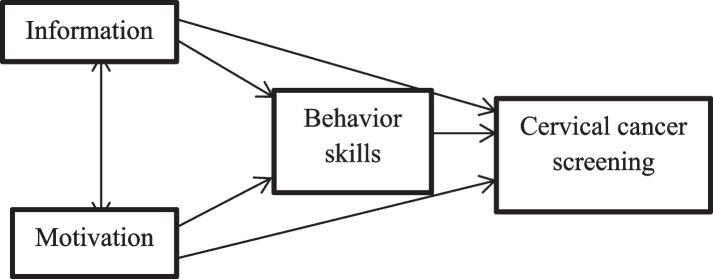
Information, motivation and behavioral skills model

 The information-motivation-behavioral skills (IMB) model is a method for understanding the social and psychological factors influencing health-related behavior. This model also determines and predicts health behavior changes [22]. Behavioral change requires specific information, motivation, and behavioral skills, according to the IMB model (Fig. 1).

This model’s components are essential for predicting and promoting healthy behavior. The IMB model is designed in a simple way to comprehend and intended to meet the needs of the public to improve their health [23].

According to the IMB model, breast cancer screening adherence is contingent on the levels of information, motivation, and behavioral skills, and there is a correlation between having information about the age of initiation and time of screening repetition, risk factors, and symptoms of cancer, family members’ support, and behavioral skills with screening [24]. According to studies, cervical cancer screening is also associated with knowledge, awareness, the husband’s support and approval, recommendations from acquaintances, friends, and family, recommendations from doctors and health personnel, previous Pap smear results, and screening history [25–30].

Even though the IMB model has been examined in various health fields, including breast cancer screening, self-care of diabetes, prevention of sexually-transmitted diseases, condom use, HIV prevention, adherence to AIDS treatment, and tuberculosis infection control [21, 22, 24, 31–40], little is known about its use in cervical cancer screening [41]. In addition, despite the fact screening can prevent cervical cancer, some women do not undergo it regularly, and cervical cancer is still considered a global health concern, mainly in developing countries [10, 17, 42–44]. Therefore, the purpose of the present study was to determine whether the information-motivation-behavioral skills (IMB) model can predict cervical cancer screening.

## Method

The current study employed a cross-sectional design and included 20-60-year-old women who visited health centers and gynecological clinics in Isfahan, Iran, between October 2020 and May 2021. The sample size was calculated using article related to analysis which is mentioned five or ten observations per estimated parameter [45]. The study enrolled a total of 310 women eligible for cervical cancer screening.

In the present study, comprehensive health centers and gynecological clinics of hospitals were randomly selected by lot. Six community health centers were selected randomly from all centers, and the Beheshti gynecological clinic was chosen randomly from two clinics of hospitals in Isfahan. Women who met the inclusion criteria were enrolled in the study using convenience sampling. The inclusion criteria included possessing an Iranian nationality, being married, aged between 20 and 60 years, and providing consent to participate in the study.

Women were recruited when they attended the centers and clinic. This was when they were approached by the researcher. Before they entered the research, the necessary information about the research was provided to them and informed consent was obtained from them. The participants were assured that their names will remain anonymous.

### Measures

The first questionnaire included demographic information (age, education level, occupation, and income) and details about the Pap smear test. So that a single question was used to evaluate cervical cancer screening (do you regularly receive a pap smear?). The question was scored using a Likert scale (1: never, 2: rarely, 3: occasionally, 4: always). Furthermore, three questionnaires including cervical cancer screening information questionnaire, cervical cancer screening motivation questionnaire and cervical cancer screening behavioral skills questionnaire were used.

### Cervical cancer screening information questionnaire

This questionnaire was developed by the researchers and contained 17 questions regarding cervical cancer risk factors, cervical cancer symptoms, cervical cancer prevention, screening initiation and termination time, Pap smear test location, and Pap smear test results. The questions were formulated where respondents could choose between “correct,” “incorrect,” and “I don’t know.’

### Cervical cancer screening motivation questionnaire

This questionnaire was developed by the researchers and consisted of three questions regarding personal motivation (Easy to perform pap smear, the importance of health and increasing survival) and three on social motivation (Recommendation to perform pap smear by husband, family and health care providers) to undergo the Pap smear test. The questions were scored using a 5-point Likert scale (1: strongly disagree to 5: strongly agree).

### Cervical cancer screening behavioral skills questionnaire

This questionnaire was developed by the researchers and included four questions regarding behavioral skills for Pap smear (planning and time management, overcoming fear, spending money, and visiting service centers). The questions were scored using a 5-point Likert scale (1: strongly disagree to 5: strongly agree).

### Validity and reliability of the tool

The questionnaires were distributed to 13 faculty members from the Department of Midwifery and Reproductive Health and Health Education and Promotion to assess the qualitative content’s validity. To this end, expert feedback was gained on the content, grammar, and appropriate expressions. Two methods were used to examine the content validity: the content validity ratio (CVR) and the content validity index (CVI). CVR was calculated based on the content validity ratio formula as fallows.

$$\left(\mathrm{Ne}-\mathrm N/2\right)/\left(\mathrm N/2\right),\;\mathrm{Ne}\:=\:\mathrm{number}\;\mathrm{of}\;\mathrm{essentials}\;\mathrm{for}\;\mathrm{an}\;\mathrm{item}\;\mathrm{and}\;\mathrm N=\mathrm{number}\;\mathrm{of}\;\mathrm{experts}$$

To calculate CVR, 13 experts were asked to rate the necessity of each item on a 3-point Likert scale ranging from “necessary” to “not necessary”. Given the presence of 13 experts, the CVR approval criterion was set at 0.54 for each question [46].

To examine CVI, experts’ opinions on each item’s relevance, clarity, and simplicity were calculated using a 4-point Likert scale. A question would be eliminated if its CVI was less than 0.7 [47]. Of these, totally nine items were excluded after assessment (one item from the motivation, two items from the behavioral skills and six items from the information).

Determining content validity led to the information questionnaire with 17 questions, the motivation questionnaire with 6 questions, and the behavioral skills questionnaire with 4 questions.

The reliability of the questionnaires was confirmed by calculating Cronbach’s alpha coefficients for several subjects (information [0.750], motivation [0.702], and behavioral skills [0.871]). The whole questionnaires obtained an acceptable score.

### Statistical analysis

The descriptive statistical test (frequency distribution and percentage) and multinomial logistic regression tests were conducted using SPSS 22 software. Multinomial logistic regression was used to examine the correlation between the components of the IMB model with cervical cancer screening. In the analysis, “never” was used as a reference for rarely, occasionally, and always.

AMOS 24.0 was utilized for path analysis. Maximum likelihood estimation was employed as the parameter estimation method. In addition, chi-square and comparative indices NFI, RFI, IFI, TLI, and CFI were utilized to assess the model’s fitness. The variables were comprised of four categories, which were consisted of never, rarely, occasionally and always.

## Findings

According to the findings, the majority of the research subjects (39.7%) were between the ages of 31 and 40, were homemakers (82.3%), held a high school diploma (34.5%), and had moderate income levels (64.8%) (Table 1).

**Table 1:** Demographic information of the subjects in the study

Variable		Number	Percent
Education level	Illiterate or Elementary	46	14.8
	High school	51	16.5
	Diploma	107	34.5
	College	106	34.2
Job	Housewife	255	82.3
	Employed	55	17.7
Economic status	Lower	58	18.7
	Middle	201	64.8
	Upper	51	16.5
Age	20–30 y	71	22.9
	31–40 y	123	39.7
	41–50	86	27.7
	Upper 50	30	9.7

Approximately 18.1% of the participants had never undergone routine cervical cancer screening. In addition, 24.2%, 25.5%, and 32.3% of participants reported undergoing cervical cancer screening rarely, occasionally, and frequently, respectively. The multinomial logistic regression analysis results indicated that the components of the model sufficiently predicted cervical cancer screening (*P* < 0.001) (Table 2).

**Table 2:** Multinomial logistic regression analysis IMB model with cervical cancer screening

Variable		Sig	Exp(B)	SE	B	Model fit
Rarely	Information	0.138	0.059	1.148	0.019	Adjusted R Square 0.406 X = 161.38 *P* > 0.001
	Motivation	0.110	0.061	1.116	0.073	
	Behavior skill	0.81	0.062	1.085	0.193	
Occasionally	Information	0.225	0.068	1.095	0.001	
	Motivation	0.66	0.068	0.936	0.328	
	Behavior skill	0.326	0.074	1.199	> 0.001	
Always	Information	0.218	0.073	1.078	0.003	
	Motivation	0.139	0.072	0.997	0.054	
	Behavior skill	0.513	0.084	1.417	> 0.001	

Never regular cervical cancer screening (Reference)

**Fig. 2:**
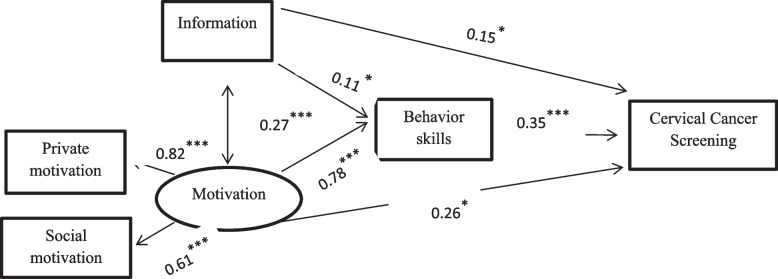
Prediction cervical cancer screening based on IMB model amos software. Chi-square = 6.89. Degrees of freedom = 2. Probability level = .032. ^*^. *P*<0/05. ***. *P*<0/001

Path analysis indicated that the model had an acceptable fit (CMIN = 6.89, df = 2, *P* = 0.032) (RFI = 0.926, IFI = 0.990, TLI = 0.949, CFI = 0.990, NFI = 0.986). The results indicated that information (β = 0.05, *P* = 0.002), motivation (β = 0.187, *P* = 0.026), and behavioral skills (β = 0.95, *P* < 0.001) has a direct effect on regular cervical cancer screening (Table 3). Furthermore, information (β = 0.137, *P* = 0.024) and motivation (β = 2.062, *P* < 0.001) had a direct effect on behavioral skills. Among the factors of information, motivation, and behavioral skills, behavioral skills had the most significant direct effect on regular cervical cancer screening (Fig. 2; Table 3).

**Table 3:** Path coefficients and the variance in IMB constructs explained

Path coefficients	Β	S.E	C.R	P
Information → Cervical Cancer Screening	0.050	0.016	3.140	0.002
Motivation → Cervical Cancer Screening	0.187	0.084	2.230	0.026
Behavior skills → Cervical Cancer Screening	0.095	0.029	3.297	< 0.001
Information → Behavior skills	0.137	0.060	2.363	0.024
Motivation → Behavior skills	2.062	0.223	9.226	< 0.001
Information ↔ Motivation	1.342	0.358	3.751	< 0.001

## Discussion

The current study sought to investigate the prediction of cervical cancer screening using the IMB model. According to the findings, the IMB model could predict cervical cancer screening in women. In the current study, the components of the IMB model predicted regular cervical cancer screening. To this end, a regular cervical cancer screening was found to correlate significantly with information, motivation, and behavioral skills.

According to studies, there are relationships between cancer screening information and motivation for women [48, 49]. Vinarti et al. [50] reported that motivation was related to cervical cancer screening. Suls et al. claimed that information determined health behavior and influenced the performance of preventive behavior. Consequently, information alone may serve as a predictor of behavior [23].

In addition, motivation is a factor that can influence the promotion of health-related behaviors. It includes personal motivation, which includes belief and attitude toward a particular health behavior, and social motivation, which includes perceived social support or social norms for engaging in the behavior [51]. People typically seek health improvement and information acquisition due to personal and social motivations [23].

Gu et al. [52] regarded previous screening experience and beliefs about cervical cancer screening as crucial factors in fostering future motivation for screening. Kalichman et al. [37] also reported a significant association between motivation and AIDS prevention behaviors. These findings were consistent with those of the present study. Moreover, increasing knowledge increased their motivation to undergo cervical cancer screening [41]. The results of the current study indicated a correlation between information and motivation regarding cervical cancer screening. According to Gao et al., motivation and information may have reciprocal effects on diabetes self-care [35].

Individuals with more information are more motivated to engage in healthy behaviors. In addition, those with greater motivation seek to expand their information on health-related behaviors [24]. In several studies, researchers have reported that individuals must possess the requisite behavioral skills to change their health-centered behaviors [22, 24, 33]. In the present study, behavioral skills had the greatest direct influence on regular Pap smear testing.

Behavioral skills are required for engaging in particular health behavior. Increasing behavioral skills adjusts the behavior of individuals and improves their health. In the IMB model, these skills emphasize increased objective skills and perceived self-efficacy to perform health behaviors [51]. According to several studies, information and motivation affect the performance of certain health behaviors by fostering behavioral skills [21, 36, 41]. Misevich et al. [24] found a correlation between information and motivation and the behavioral skills required to perform monthly breast examinations. Mayberry et al. [34] also reported that information and motivation were associated with diabetes treatment adherence behavioral skills. These findings were consistent with those of the present study. This model indicates that changes in behavior largely result from changes in behavioral skills, primarily the result of changes in information and motivation [36].

Notably, the IMB model does not account for all determinants of health behavior performance, including mental health, access to medical services, medical history, norms, and cultural factors [24, 53]. Based on the results of the present study, this model can provide a comprehensive framework for predicting cervical cancer screening in women.

To the best of the authors’ knowledge, the present study was the first to be conducted based on the IMB model, which examined the roles of information, motivation, and behavioral skills in cervical cancer screening. Despite research on the roles of information and motivation variables in cervical cancer screening [48, 49], no research has been conducted on the roles of behavioral skills as direct and mediating variables in cervical cancer screening.

Even though the results of this study supported the use of the IMB model for predicting cervical cancer screening, it was not possible to investigate other causal relationships influencing cervical cancer screening in this study.

There is another issue that should be mentioned. The term virginity as the absence of sex before marriage is considered as a value in some societies [54]. Due to the prevailing culture of the society, only married women were included in the study. It is one of the limitations of present study which may affect the generalizability of the results. Another limitation related to self-reported Pap smear examinations in women.

## Conclusion

According to the findings of this study, the IMB model provided an appropriate framework for predicting cervical cancer screening. Information and motivation were associated with behavioral skills in cervical cancer screening, and behavioral skills were the most significant predictors of screening. Consequently, it is possible to improve cervical cancer screening in women by designing and implementing necessary interventions based on this model, particularly by improving behavioral skills.

### Supplementary Information

**Supplementary Material 1.**

## Data Availability

The datasets generated during the current study are available from the corresponding author on reasonable request.

## Abbreviations

IMB: Information–motivation–behavioral skills model
CVI: Content validity index
CVR: Content validity ratio
CMIN: χ2
RFI: Relative fit index
IFI: Incremental fit index
TLI: Tucker-Lewis index
CFI: Confirmatory factor analytic
NFI: Normed fit index
DF: Degree of freedom
