# Supplementary material for: Prediction of cervical cancer screening: application of the information-motivation-behavioral skills model
Source: BMC Cancer. 2024 Mar 19;24:351. doi: 10.1186/s12885-024-12098-9 (PMC10949627; doi:10.1186/s12885-024-12098-9)
Supplement: Supplementary file 1 — Supplementary Material 1. [file 12885_2024_12098_MOESM1_ESM.docx]

**Cervical cancer screening behavioral skills questionnaire**

|  |  | **Strongly‎ agree** | **Agree** | **No opinion** | **Disagree** | **Strongly‎ disagree** |
| --- | --- | --- | --- | --- | --- | --- |
| 1 | Although I must plan to find centers where do Pap smear test, I do it. |  |  |  |  |  |
| 2 | I am able to arrange a time for doing regular Pap smear. |  |  |  |  |  |
| 3 | I am able to overcome the fear of doing Pap smear test. |  |  |  |  |  |
| 4 | I have the possibility of cost management to do Pap smear test. |  |  |  |  |  |

**Cervical cancer screening motivation questionnaire**

|  | **I am having a Pap smear test because........** | **Strongly‎ agree** | **Agree** | **No opinion** | **Disagree** | **Strongly‎ disagree** |
| --- | --- | --- | --- | --- | --- | --- |
| 1 | Early detection of cervical cancer can increase my chances of survival |  |  |  |  |  |
| 2 | My health is very important to me |  |  |  |  |  |
| 3 | My husband encourages me to do a Pap smear |  |  |  |  |  |
| 4 | My family members and friends recommend me to do Pap smear test |  |  |  |  |  |
| 5 | Doctors, midwives or other healthcare providers recommend a Pap smear test |  |  |  |  |  |
| 6 | Pap smear test is easy to do |  |  |  |  |  |

**Cervical cancer screening information questionnaire**

|  |  | **Correct** | **Incorrect** | **I don't know** |
| --- | --- | --- | --- | --- |
| 1 | Having sex under the age of 16 increases the risk of cervical cancer. |  |  |  |
| 2 | Having multiple sexual partners increases the risk of cervical cancer |  |  |  |
| 3 | Women who have more given birth are less at risk of cervical cancer |  |  |  |
| 4 | Sexually transmitted infections increases the risk of cervical cancer |  |  |  |
| 5 | Using a condom prevents cervical cancer |  |  |  |
| 6 | One of the early detection methods of cervical cancer is pap smear test |  |  |  |
| 7 | Smoking does not play a role in increasing the risk of cervical cancer |  |  |  |
| 8 | Obesity increases the risk of cervical cancer |  |  |  |
| 9 | If the Pap smear test result is normal, it should be repeated every 3 years |  |  |  |
| 10 | Pap smear test is not necessary for all married women |  |  |  |
| 11 | If women have sex from the age of 21, they should have a Pap smear test |  |  |  |
| 12 | With the onset of menopause, there is no need to do a Pap smear |  |  |  |
| 13 | Pap smear test can be done during menstruation |  |  |  |
| 14 | If the Pap smear test result is abnormal, the cause should be investigated |  |  |  |
| 15 | To perform a Pap smear test, you should see a gynecologist or a midwife |  |  |  |
| 16 | Bleeding or spotting after intercourse can be a symptom of cervical cancer |  |  |  |
| 17 | Pain during intercourse is not a symptom of cervical cancer |  |  |  |
